# Characterization of gut microbiota signatures in Indian preterm infants with necrotizing enterocolitis: a shotgun metagenomic approach

**DOI:** 10.3389/fcimb.2025.1649384

**Published:** 2025-09-11

**Authors:** Prabavathi Devarajalu, Savita Verma Attri, Jogender Kumar, Sourabh Dutta, Jayakanthan Kabeerdoss

**Affiliations:** ^1^ Pediatric Biochemistry Unit, Department of Pediatrics, Post Graduate Institute of Medical Education & Research (PGIMER), Chandigarh, India; ^2^ Newborn Unit, Department of Pediatrics, Post Graduate Institute of Medical Education & Research (PGIMER), Chandigarh, India

**Keywords:** Necrotizing enterocolitis, gut microbiota, preterm infants, shotgun metagenomics, LPS O-antigen, TLR4, type IV secretion system (T4SS), India

## Abstract

**Introduction:**

Necrotizing enterocolitis (NEC) is an inflammatory bowel disease that primarily affects preterm infants. Predisposing risk factors for NEC include prematurity, formula feeding, anemia, and sepsis. To date, no studies have investigated the gut microbiota of preterm infants with NEC in India.

**Method:**

In the current study, shotgun metagenomic sequencing was performed on fecal samples from premature infants with NEC and healthy preterm infants (n = 24). Sequencing was conducted using the NovaSeq X Plus platform, generating 2 × 150 bp paired-end reads. The infants were matched based on gestational age and postnatal age.

**Result:**

The median time to NEC diagnosis was 9 days (range: 1–30 days). Taxonomic analysis revealed a high prevalence of *Enterobacteriaceae* at the family level, with the genera *Klebsiella* and *Escherichia* particularly prominent in neonates with NEC. No statistically significant differences in alpha or beta diversity were observed between stool samples from infants with and without NEC. Linear regression analysis demonstrated that *Enterobacteriaceae* were significantly more abundant in stool samples from infants with NEC than without NEC (q < 0.05). Differential abundance analysis using Linear Discriminant Analysis Effect Size (LEfSe) identified *Klebsiella pneumoniae* and *Escherichia coli* as enriched in the gut microbiota of preterm infants with NEC. Functional analysis revealed an increase in genes associated with lipopolysaccharide (LPS) O-antigen, the type IV secretion system (T4SS), the L-rhamnose pathway, quorum sensing, and iron transporters, including ABC transporters, in stool samples from infants with NEC.

**Conclusion:**

The high prevalence of *Enterobacteriaceae* and enrichment of LPS O-antigen and T4SS genes may be associated with NEC in Indian preterm infants.

## Introduction

Necrotizing enterocolitis (NEC) is a devastating inflammatory disease that significantly contributes to morbidity and mortality in preterm infants. The incidence of NEC is approximately 7% (95% CI: 6–8%) among very low birth weight (VLBW) infants (Alsaied et al., 2020). Risk factors for NEC, such as formula feeding, maternal and neonatal antibiotic exposure, and premature birth, have been shown to induce alterations in gut microbiota (Thänert et al., 2020). The immature intestines of preterm infants, when exposed to inappropriate bacterial colonization alongside a hyperactivated immune system, may drive the development of NEC (Mara et al., 2018). Dysbiosis of the gut microbiota has been observed in individuals with NEC even beyond five years of age (Magnusson et al., 2024). The severity of dysbiosis correlates with the stage of NEC and is more pronounced in patients who undergo surgical treatment (Magnusson et al., 2024). These findings demonstrate how the sequelae of NEC lead to persistent perturbations in the gut microbiota of affected children.

Despite two decades of extensive research using next-generation sequencing techniques, the specific microbial communities responsible for the development of NEC have not yet been identified. Several factors contribute to this challenge, including heterogeneity among studies, such as variations in the timing of sample collection, duration of clinical presentations, and differences in sequencing methods—ranging from targeting variable regions of the 16S rRNA gene to whole-genome bacterial sequencing. Additionally, the gut microbiota of preterm infants is influenced by environmental factors related to hospital admission, antibiotic use, feeding methods, medications, and other supportive interventions (Thänert et al., 2024). During hospitalization, feed intolerance, sepsis, and other comorbidities commonly co-occur with NEC, further contributing to alterations in the gut microbiota. Probiotics have been shown to restore dysbiosis in infants, thereby reducing the incidence of NEC (Samara et al., 2022). Disruptions in the gut microbiota during the early weeks of life in preterm infants are associated with an increased risk of NEC.

Several studies conducted in high-income countries have established an association between the microbiome and NEC (Thänert et al., 2020; Tarracchini et al., 2021); however, data from low- and middle-income countries remain scarce. Investigating the gut microbiota is valuable not only for identifying microbial biomarkers but also for developing bacterial-mediated therapies, such as fecal microbiota transplantation and bacteriophage treatment. In this study, we employed a shotgun metagenomic approach to characterize the gut microbiome and identify functional metabolic pathways as the primary and secondary outcomes in fecal samples from preterm infants with NEC.

## Methods

### Participant recruitment

This prospective observational cohort study was conducted from September 2022 to January 2025 at a tertiary care neonatal unit in Northern India. Preterm infants born between 26 and 32 weeks of gestation, with a birth weight of less than 1500 grams, and admitted to the NICU were recruited. Participants’ demographic information and clinical details were collected prospectively until discharge or death using a standardized proforma. NEC was diagnosed according to the Bell staging criteria and classified as cases. Healthy preterm infants matched for gestational and postnatal age were recruited as controls. The study was approved by the Institutional Ethics Committee (IEC) of the Postgraduate Institute of Medical Education and Research (PGIMER), Chandigarh, and was conducted in accordance with the Declaration of Helsinki.

### Sample collection

The fecal samples from infants were collected in sterile containers during the first month of life as described in previous studies (Devarajalu et al., 2024; Devarajalu et al., 2025). Fecal samples from control subjects were collected on days of life matched to those of the NEC group. Participants were recruited after obtaining written informed consent from one of the parents. Stool samples were stored in an ultra-low temperature freezer (-80 °C) until further processing.

### Whole genome sequencing

Genomic DNA was isolated from approximately 100 milligrams of fecal specimens using the QIAamp PowerFecal Pro DNA Kit (Qiagen Inc., Germany). The purity of the extracted DNA was assessed by 1% agarose gel electrophoresis. DNA concentration was determined using the Qubit DNA High Sensitivity (HS) assay (Invitrogen). A paired-end DNA library was prepared using the Twist EF Library Prep Kit (Illumina, Inc., USA). Briefly, the DNA was first fragmented to the desired size, then end-repaired and mono-adenylated at the 3’ end in a single enzymatic reaction. Next, adapters were ligated to the DNA fragments using a T4 DNA ligase-based reaction. Following ligation, the fragments were prepared as substrates for PCR-based indexing in the subsequent step. During PCR, barcodes were incorporated using unique primers for each sample, enabling multiplexing. All prepared libraries were assessed for fragment distribution using a 5300 Fragment Analyzer system (Agilent Technologies, Inc., CA, United States). The resulting libraries were pooled and diluted to achieve optimal loading concentrations. Finally, the pooled libraries were loaded onto a NovaSeq X Plus system (Illumina, Inc., CA, United States) to generate 150 bp paired-end reads. The average read count of the samples is 22.8 million. Sequencing statistics are shown in **Supplementary Table 1**.

### Sequencing and mapping

The adapter sequences were trimmed using the fastq-mcf tool (version 1.04.803). The trimmed reads were then aligned to the human genome (hg19) using BWA (version 0.7.12) to remove human contamination. The remaining unaligned reads were *de novo* assembled with MEGAHIT (version 1.2.9). The resulting assembled genome was used for open reading frame (ORF) prediction and annotation with Prodigal (version 2.6.3). Taxonomic classification was performed using datasets from the National Center for Biotechnology Information (NCBI), and functional pathways were identified from the predicted ORFs using SEED protein classification in MEGAN 6 (Huson et al., 2016).

### Microbiome composition and statistical analysis

Alpha diversity metrics, including observed counts and the Shannon index, were calculated using the Phyloseq package (McMurdie and Holmes, 2013). Differences in alpha diversity between groups were assessed using the Wilcoxon rank-sum test. Beta diversity metrics were computed with the Vegan package. A permutational analysis of variance (PERMANOVA) was performed using the adonis2 function from the pairwise package to identify variation between groups by fitting Bray-Curtis and Jaccard distance matrices separately, incorporating covariates, with 999 permutations (Oksanen et al., 2001).

Differential bacterial abundance between variables was analyzed using Linear Discriminant Analysis (LDA) Effect Size (LEfSe) with an LDA threshold of 4, implemented through the MicrobiomeMarker package (Cao et al., 2022). Additionally, a linear regression model employing the LinDA and MaAsLin2 packages was used to identify taxa and functional pathways associated with necrotizing enterocolitis (NEC) (Mallick et al., 2021; Zhou et al., 2022). Multiple testing corrections, Benjamini-Hochberg (BH) was applied, with a significance threshold of q < 0.05. Welch’s t-test was conducted for differential functional analysis using STAMP software (Parks et al., 2014). Correlation and network analyses were performed using the Network Construction and Comparison for Microbiome Data (NetCoMi) package (Peschel et al., 2021). All statistical analyses were conducted using R software (version 4.4.2), and figures were generated using the ggplot2, pheatmap, and MicrobiomeStat packages.

## Results

### Baseline demographic characteristics

The baseline demographic information is presented in **Table 1**. The NEC and control groups were matched for gestational and postnatal ages. One sample (NEC) was excluded from downstream analysis due to low sequencing read quality.

**Table 1:** Demographic details of subjects.

Variables	Control n=12	NEC n=12	P value
Gestational age (week), mean ± SD	30.5 ± 2.17	30.4 ± 1.75	0.829
Sex (male/female)	6/6	6/6	1
Birth weight (g), mean ± SD	1235 ± 311	1259 ± 289.4	0.978
No. (%) of mothers with PPROM	3 (25%)	2 (16.67%)	0.615
Mode of delivery (Vaginal/LSCS)	6/6 (50/50%)	6/6 (50/50%)	1
No. (%) of mothers with preeclampsia	4 (33.3%)	1 (8.3%)	0.314
No. (%) of mothers received antenatal corticosteroids	12 (100%)	11 (92%)	0.307
APGAR score at 1 min median (IQR)	7 (6-9)	6 (5-9)	0.922
APGAR score at 5 min median (IQR)	8 (7-9)	7 (6-9)	0.493
No. (%) of neonates receiving antibiotic	12 (100%)	12 (100%)	1
Day of life on which stool sample was collected	6 (3-20)	6 (3-20)	1
Age of diagnosis of NEC (days)	NA	9 (3-30)	NA
NEC associated deaths	NA	2 (16.7%)	NA

PPROM, Preterm Prelabor Rupture of Membranes; LSCS, Lower Segment Cesarean Section; SD, Standard deviation; IQR, Interquartile range; NA, Not applicable.

### Microbial richness and diversity

**Figure 1:**
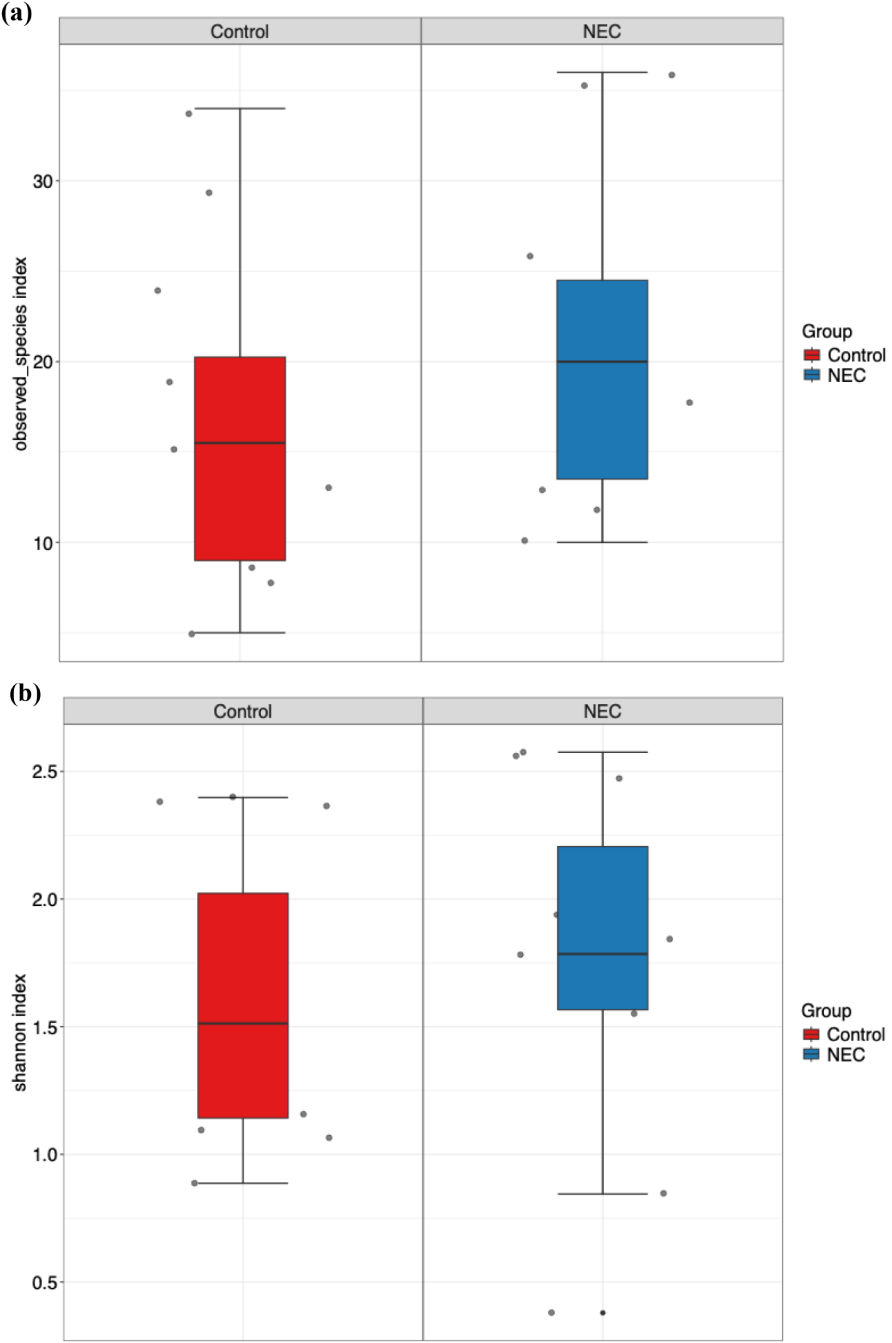
Box and whisker plot represents alpha diversity index **(a)** observed ASVs and **(b)** Shannon index for NEC and control groups.

**Figure 2:**
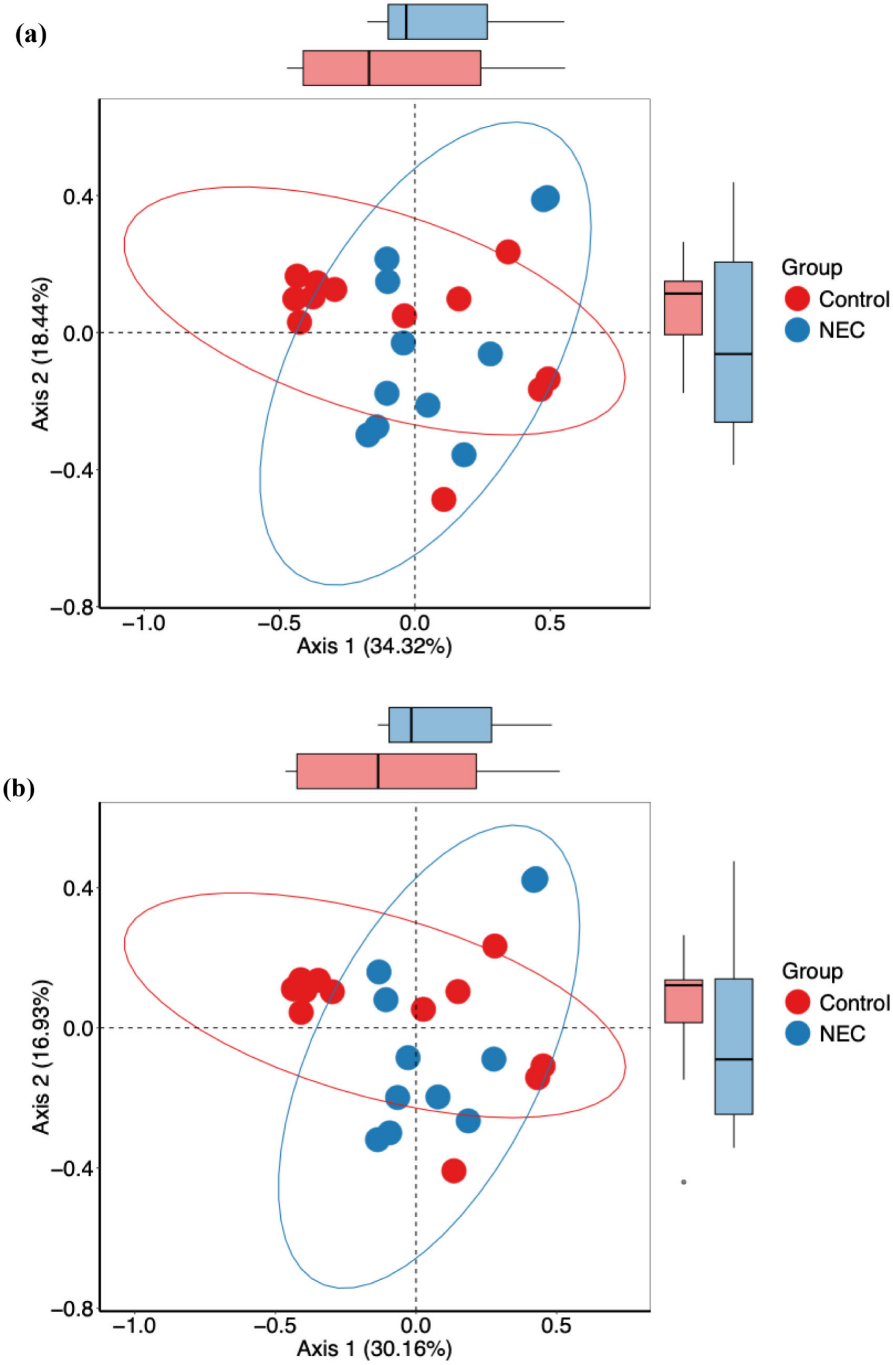
Principal coordinate analysis (PCoA) plots showing the beta diversity with **(a)** Bray-Curtis and **(b)** Jaccard measures for NEC and control groups.

Microbial richness, assessed using alpha diversity, showed no significant differences in observed operational taxonomic units (OTUs) and Shannon index between infants with NEC and the control group (**Figure 1**). Alpha diversity indices did not differ significantly when analyzed against other covariates, including mode of delivery (vaginal vs. LSCS), sex (male vs. female), birth weight (<1000 g vs. >1000 g), and gestational age (<28 weeks vs. 28–32 weeks). Furthermore, no statistically significant differences or effect size variations were observed between NEC and control groups for beta diversity, as measured by both Bray-Curtis and Jaccard distance metrics (**Figure 2**). PERMANOVA analysis revealed that mode of delivery accounted for a greater variance in beta diversity than other covariates within our cohort (**Table 2**).

**Table 2:** PERMANOVA multivariate analysis performed based on Bray–Curtis dissimilarity distance.

Variables	Df	Sums of sqs	R2	F.model	Pr (>F)
Group (NEC Vs Control)	1	0.4336	0.0565	1.2584	0.217
Preterm (Extreme Vs Very)	1	0.2623	0.0342	0.7437	0.764
Mode of Delivery (Vaginal Vs LSCS)	1	0.6204	0.0809	1.8485	0.038
Gender (Male Vs Female)	1	0.4300	0.05607	1.2475	0.213
Birth weight (<1000 g Vs ≥ 1000 g)	1	0.3123	0.04073	0.8917	0.546

LSCS, Lower Segment Cesarean Section.

### Taxonomic composition and differential abundance

**Figure 3:**
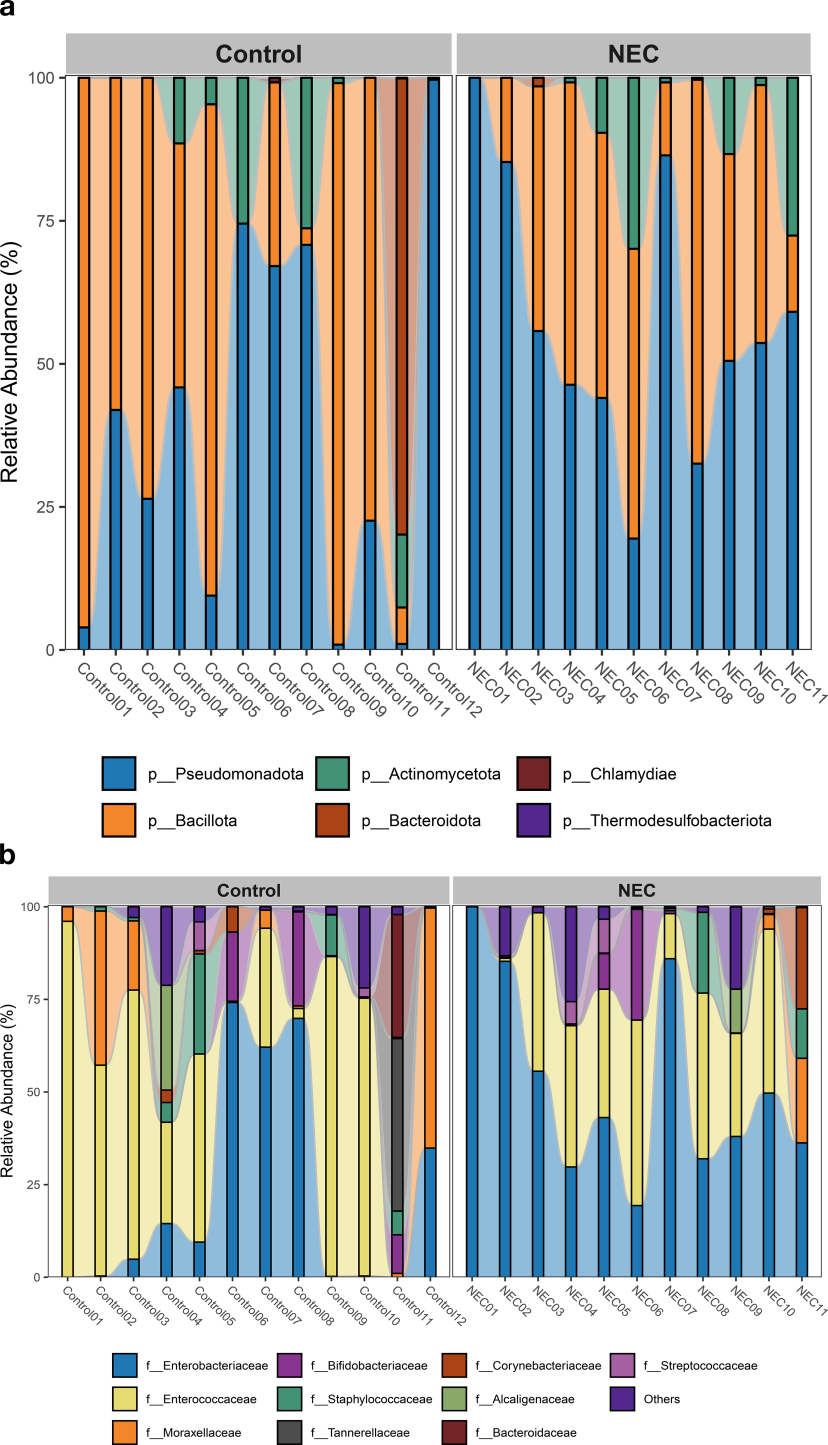
Bar plot represents taxonomic composition of both NEC and controls at **(a)** phylum and **(b)** family level.

**Figure 4:**
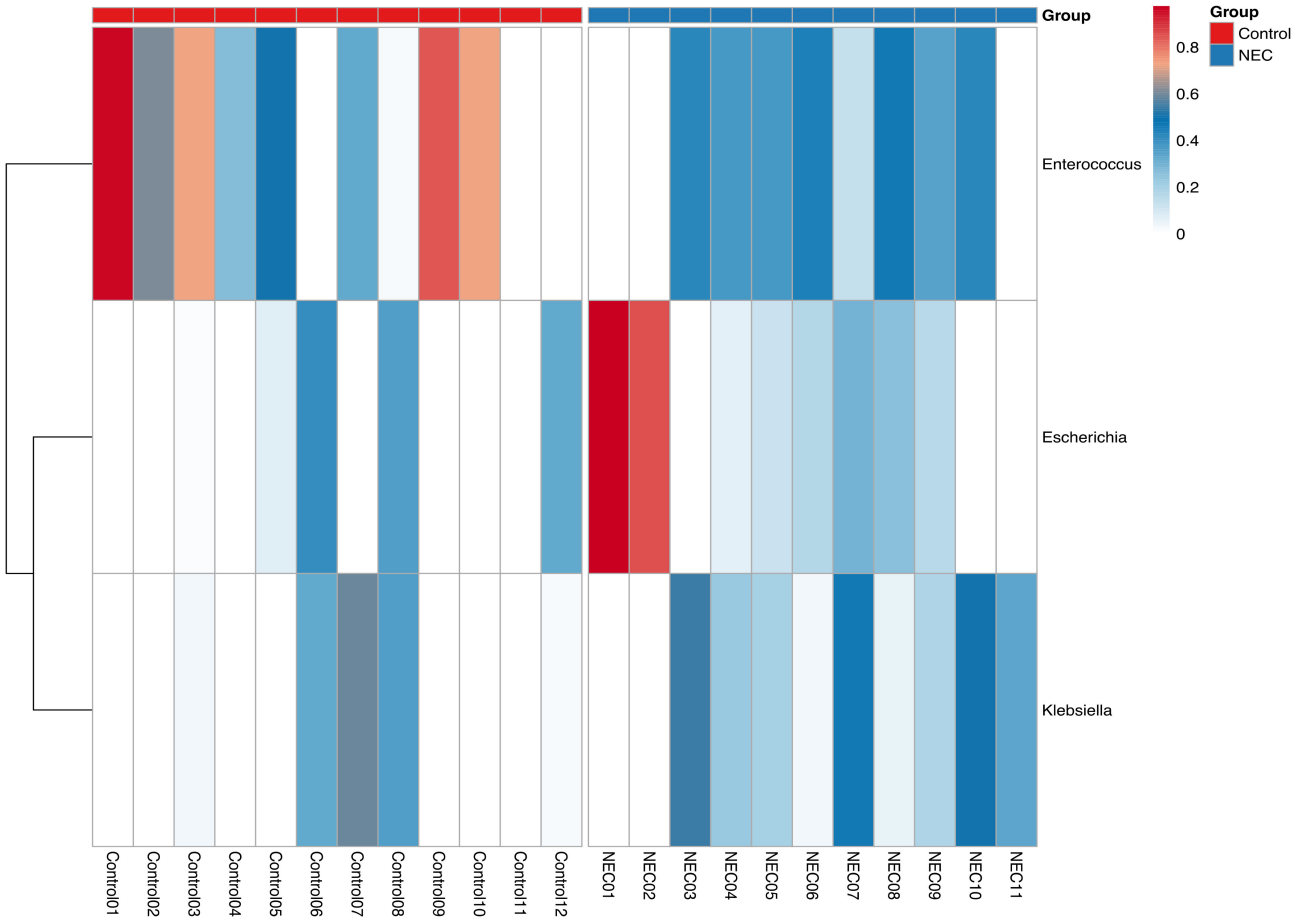
Heatmap showing three prominent genera of NEC and control groups.

**Figure 5:**
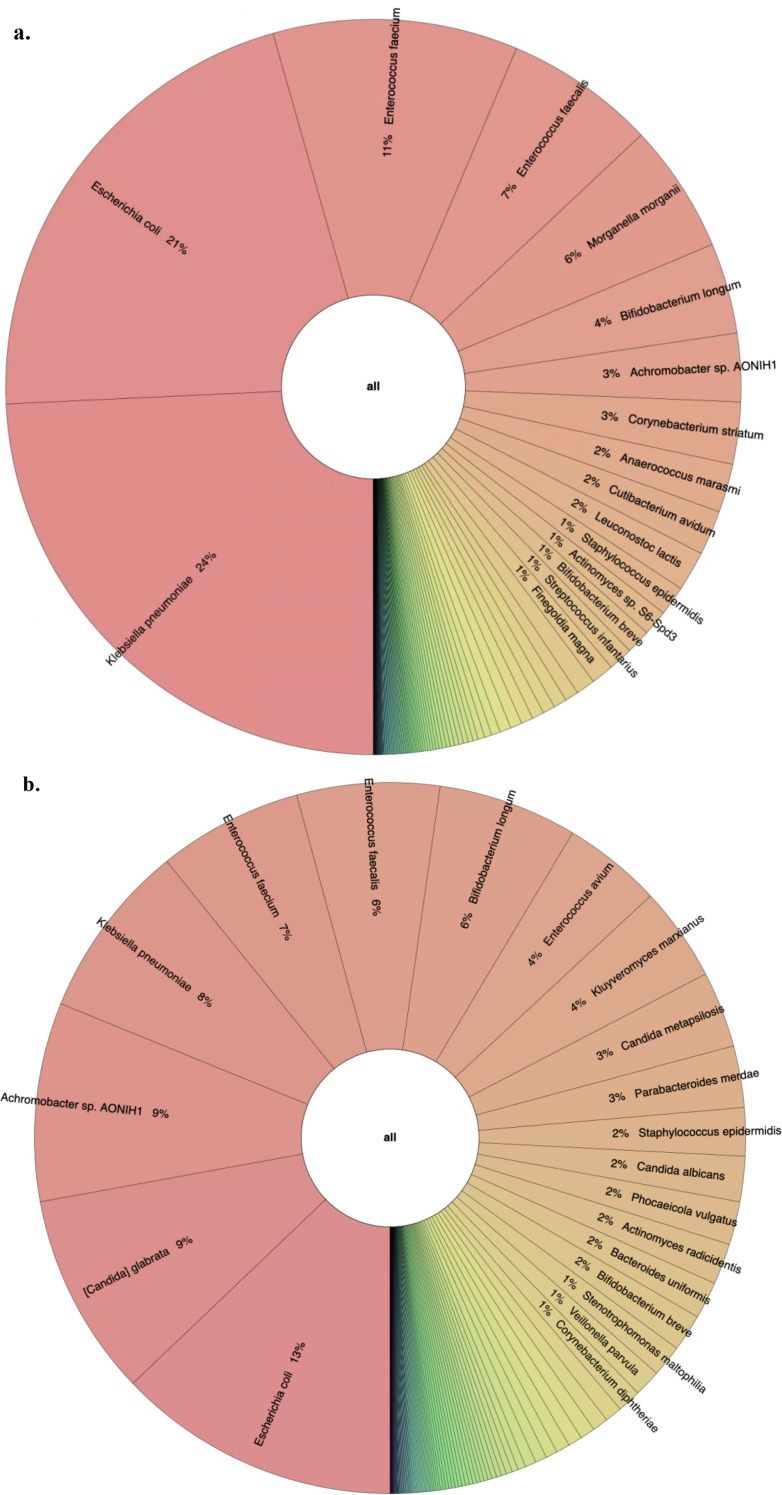
A Krona plot showing composition at species levels for NEC and controls. **(a)** NEC. **(b)** Control.

The taxonomic composition at the phylum level indicated that *Pseudomonadota, Bacillota*, and *Actinomycetota* were the predominant phyla during the first week of life. An increased abundance of *Pseudomonadota* was observed in preterm infants with NEC, accounting for 47% of the total phyla (**Figure 3a**). The two major families identified in the gut of preterm infants during the first week were *Enterobacteriaceae* and *Enterococcaceae*, which together comprised 60% of the total taxonomic families (**Figure 3b**). The abundance of *Enterobacteriaceae* was significantly higher in the gut of infants with NEC than without NEC, as demonstrated by linear regression analysis (**Table 3**). There was an increased prevalence of the genera *Escherichia* and *Klebsiella* and a decreased prevalence of *Enterococcus* in preterm infants with NEC. Linear regression analysis indicated a significant increase in the abundance of the genus *Klebsiella* in NEC cases. Heatmap analysis revealed that either *Escherichia* or *Klebsiella* was present in 82% of NEC cases compared to 33.3% of controls (**Figure 4**). Krona plots showed that the abundance of *Klebsiella pneumoniae* in the NEC and control groups was 24% and 8% of the total microbial OTUs, respectively, while *Escherichia coli* accounted for 21% in the NEC cases and 13% in controls (**Figure 5**).

**Table 3:** Linear regression analysis at family levels between NEC and control groups.

Variable	Coefficient	SE	P value	Adjusted p value	Abundance	Prevalence
*Bifidobacteriaceae*	-0.0182	0.9432	0.9847	0.9847	0.0417	0.2608
*Corynebacteriaceae*	0.2066	0.7670	0.7902	0.9659	0.01949	0.3043
*Enterobacteriaceae*	4.1053	1.2305	0.0031	0.0344	0.3660	0.9130
*Enterococcaceae*	0.07559	1.2171	0.9510	0.9847	0.3470	0.7826
*Listeriaceae*	0.1316	0.2551	0.6113	0.9453	0.0002	0.1304
*Moraxellaceae*	-3.0507	1.3559	0.0353	0.1294	0.0693	0.5217
*Staphylococcaceae*	-0.7351	1.2153	0.5517	0.9453	0.0364	0.6521
*Streptococcaceae*	0.9612	1.0025	0.3485	0.9453	0.0133	0.3478

**Figure 6:**
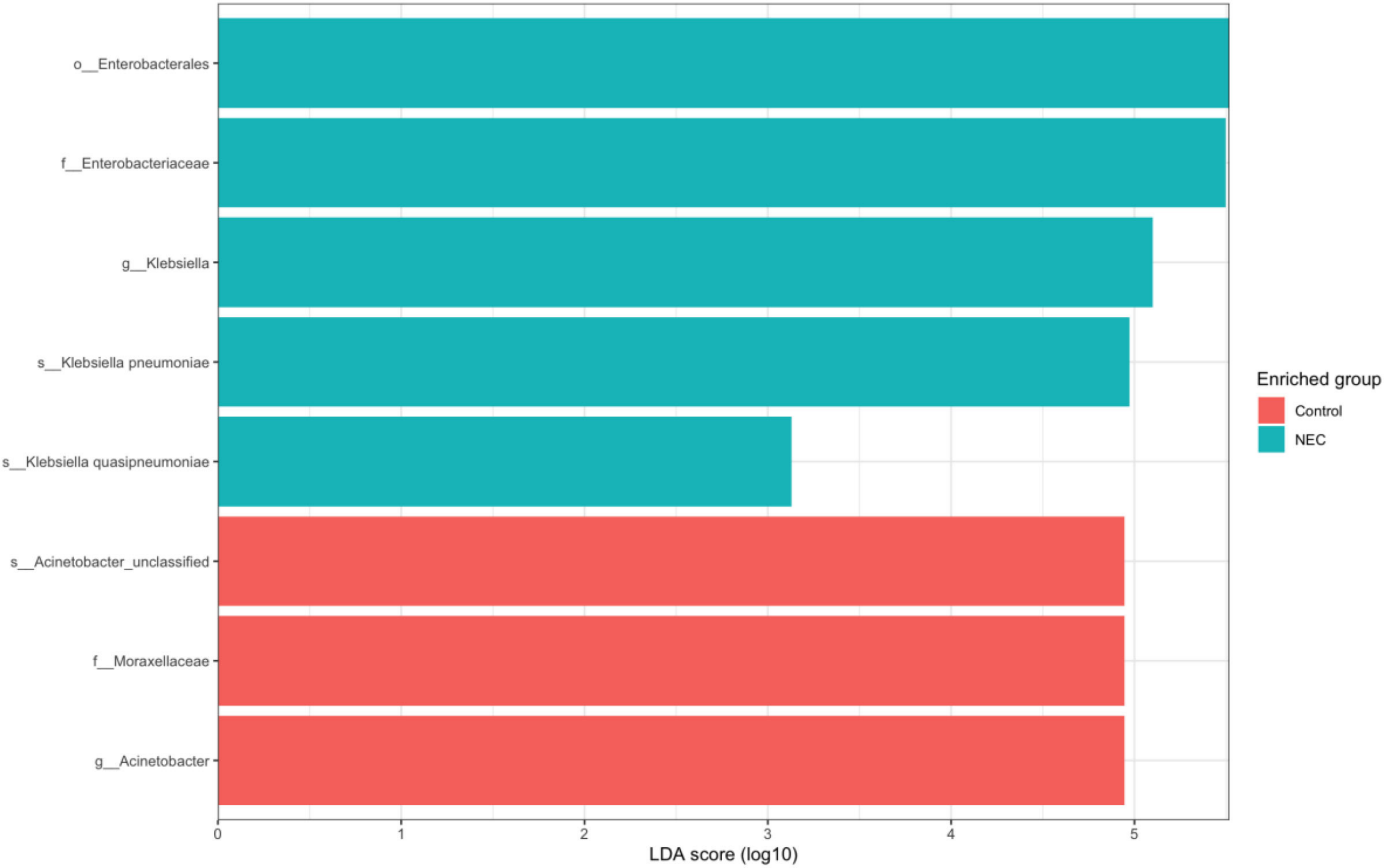
Bar stack plot showing LEfSe results of differential abundance at species levels between NEC and Controls (p < 0.05, LDA > 4).

Differential abundance analysis using LEfSe revealed that *Klebsiella pneumoniae* and *Klebsiella quasipneumoniae* were more prevalent in infants with NEC, whereas unclassified *Acinetobacter* species were more abundant in the non-NEC group (**Figure 6**).

### Correlation of microbial community

The relationships between microbial communities at the genus level were evaluated using Pearson’s correlation analysis. A strong positive correlation was observed between *Finegoldia* and *Cutibacterium* as well as between *Paraburkholderia* and *Clostridioides.* Weak positive correlations were found between *Shigella* and *Klebsiella*, and between *Escherichia* and *Klebsiella.* Additionally, weak negative correlations were noted between *Bifidobacterium* and *Enterococcus* and between *Enterobacter* and *Acinetobacter* (**Supplementary Figure 1**).

### Functional pathway analysis

**Figure 7:**
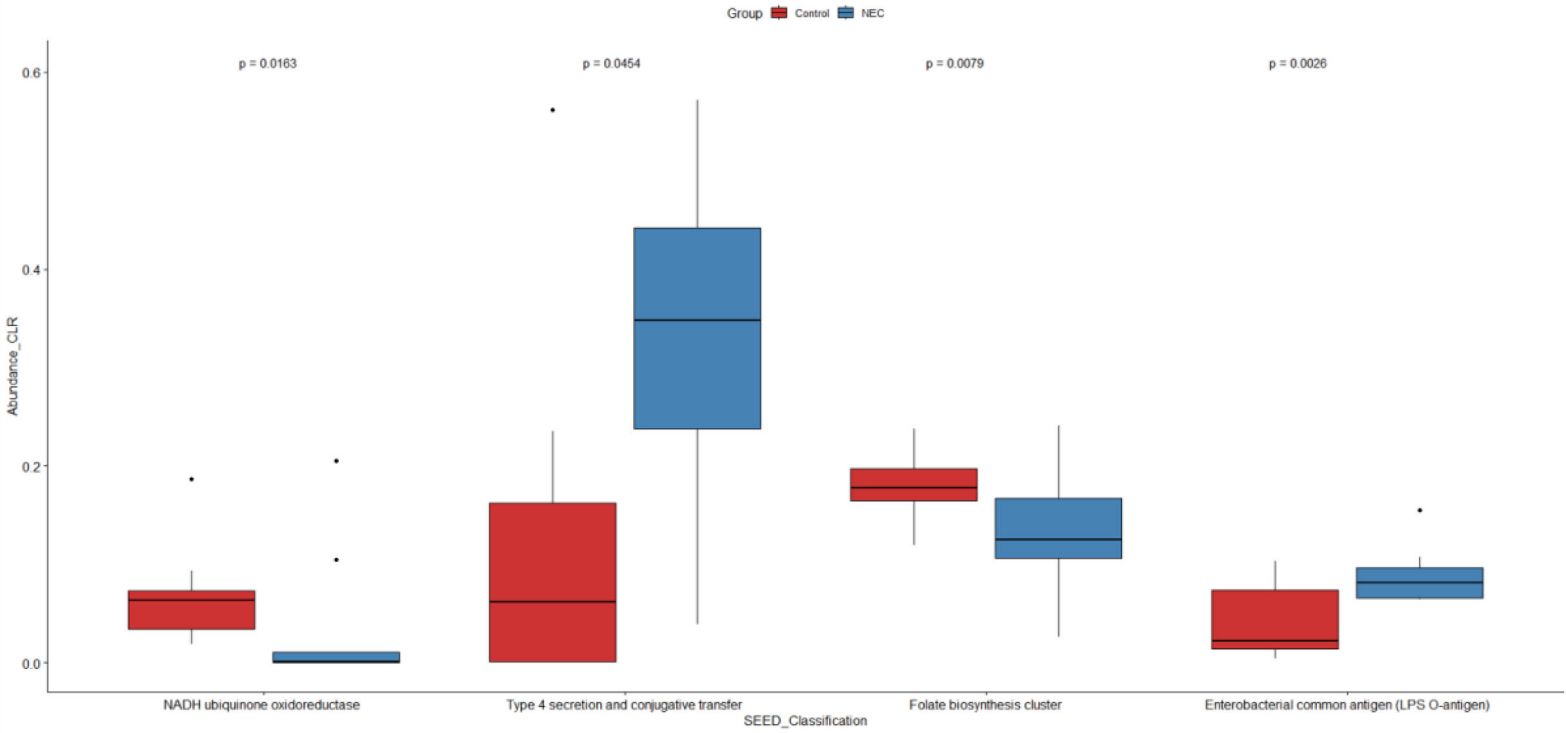
Significant functional pathways between NEC and control groups. Student t test was performed.

**Figure 8:**
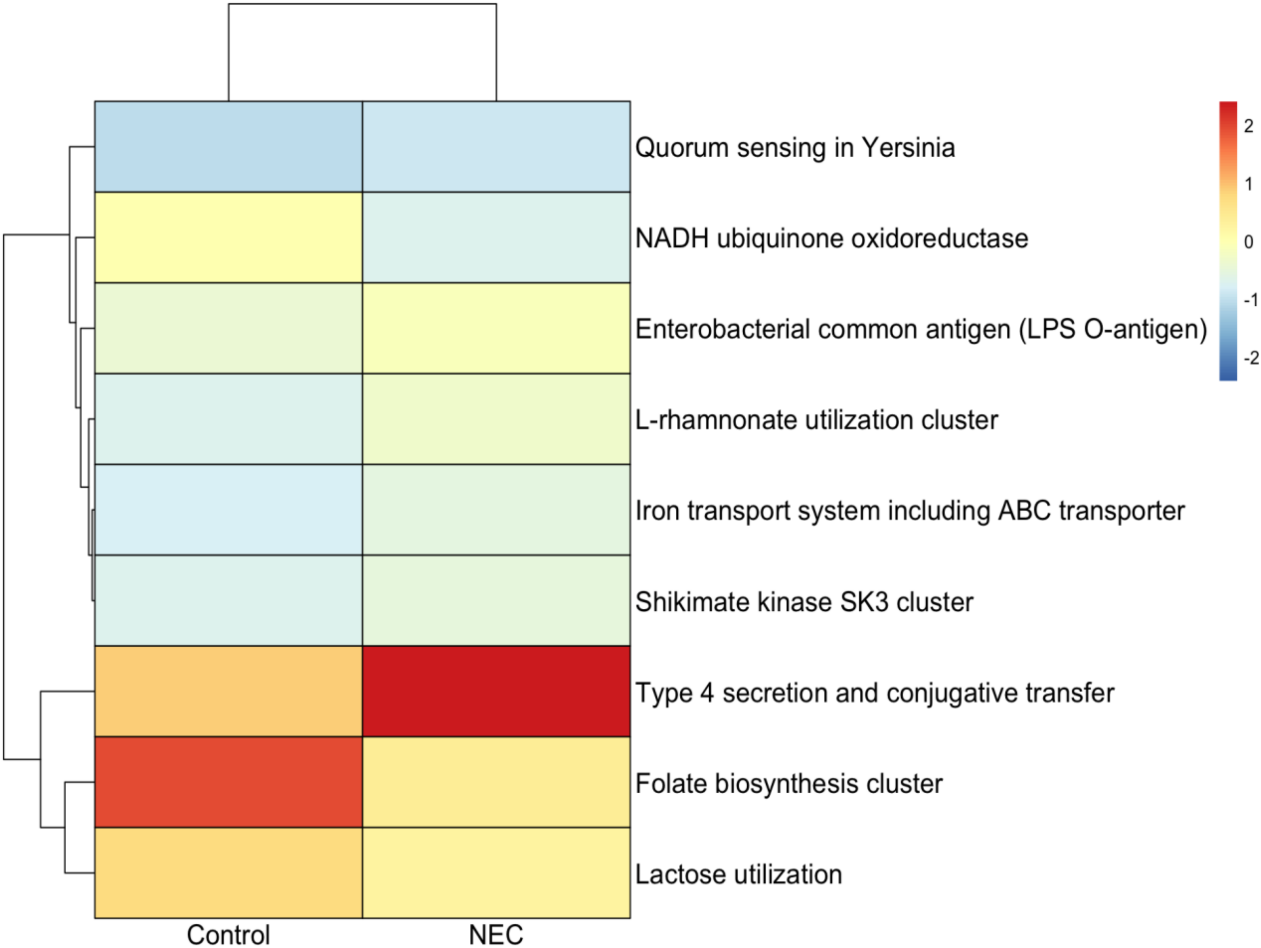
Heatmap showing differential functional pathway analysis between NEC and control groups.

Functional analysis using SEED classification revealed that pathways associated with type IV secretion systems, conjugative transfer, Enterobacterial common antigen (LPS O-antigen), quorum sensing in Yersinia, the L-rhamnose pathway, and iron transport systems—including ABC transporters and the Shikimate kinase SK3 cluster—were significantly enriched in infants with NEC compared to controls (**Figures 7**, **8**). These pathways were significant in both linear regression analysis and Welch’s t-test using the STAMP tool (**Supplementary Figure 2**). Additionally, genes involved in folate biosynthesis and lactose utilization were reduced in the NEC group compared to the control group.

## Discussion

The present study identified that *Enterobacteriaceae* were significantly more abundant in the stool samples of infants with NEC. Functional analysis revealed an increased abundance of genes involved in the biosynthesis of lipopolysaccharide (LPS) O-antigen, the type IV secretion system (T4SS), the L-rhamnose pathway and quorum sensing in the stool samples of infants with NEC. To the best of our knowledge, this is the first study to examine gut microbial communities using whole-genome shotgun sequencing in Indian preterm infants with NEC.

PERMANOVA analysis demonstrated that the mode of delivery influenced the beta diversity of the gut microbiome. However, the differential bacterial abundance identified through linear regression analysis between NEC and control groups was not affected by the mode of delivery. This is likely because the number of infants delivered vaginally and by C-section was equal in both the NEC and control groups.

The median gestational age of infants with NEC in our cohort was 30.5 ± 2.17 weeks, comparable to that reported in Western studies evaluating the gut microbiome in these infants, which showed a median gestational age of 30.1 ± 2.4 weeks (Pammi et al., 2017). No significant differences were observed between the NEC and control groups in alpha diversity indices or beta diversity measures. This finding is consistent with previous meta-analyses on the subject (Pammi et al., 2017).

Differential abundance analysis at the family level revealed elevated levels of *Enterobacteriaceae* and *Pseudomonadaceae* in the fecal samples of infants with NEC. However, the increase in *Pseudomonadaceae* did not reach statistical significance after p-value adjustment. The overrepresentation of *Enterobacteriaceae* in fecal samples from infants with NEC has been documented in studies using both culture-based and molecular techniques (Millar et al., 1992; Marolda and Valvano, 1995; Brower-Sinning et al., 2014). *Pseudomonadaceae* has also been associated with NEC and sepsis in preterm infants (Morrow et al., 2013; Wang et al., 2024). A previous study demonstrated that colonization with uropathogenic *E. coli* (UPEC) is associated with NEC-related mortality (76%) (Ward et al., 2016). In contrast, the current study found that only 21% of the infants were colonized with E. coli.

*Klebsiella pneumoniae and Klebsiella quasipneumoniae* were the dominant species identified in infants with NEC in our study. A previous study found that *K. pneumoniae* and *Klebsiella oxytoca* are associated with NEC (Paveglio et al., 2000; Thänert et al., 2024). *K. quasipneumoniae* is a recently classified subspecies of *K. pneumoniae*. Numerous studies have reported that *K. quasipneumoniae* acquires antimicrobial resistance genes (ARGs) and is associated with hospital-acquired infections (Mathers et al., 2019). *K. quasipneumoniae* has been detected in the rectal and fecal samples of preterm infants admitted to the neonatal intensive care unit (NICU) (Crellen et al., 2019; Chen et al., 2020). Previous studies showed that *Clostridium neonatale* and *Clostridium perfringens* were associated with NEC onset (Tarracchini et al., 2021). However, we did not found any association between *Clostridium* and NEC in our cohort.

Functional pathway analysis revealed that pathways associated with type IV secretion systems (T4SS), LPS O-antigen, quorum sensing in *Yersinia*, the L-rhamnose pathway, and iron transport systems including ABC transporters and the shikimate kinase SK3 cluster, were significantly increased in NEC. T4SS facilitates horizontal gene transfer among bacteria, promoting the dissemination of antibiotic resistance genes and the delivery of virulence factors. The LPS O-antigen is a conserved surface antigen found in *the Enterobacteriaceae family.* The increased abundance of *Enterobacteriaceae* in NEC samples may result in elevated levels of LPS antigens. LPS antigen-induced activation of Toll-like receptor 4 (TLR4) pathway is a well-characterized mechanism contributing to intestinal inflammation and necrosis in animal models of NEC as well as patients (Shaw et al., 2021). The L-rhamnose pathway is involved in the synthesis of LPS O-antigen in Gram-negative bacteria, including *Enterobacteriaceae* (Marolda and Valvano, 1995). The shikimate kinase SK3 pathway is crucial for the synthesis of aromatic amino acids, such as tyrosine and tryptophan, which are essential for bacterial growth and survival, including in *E. coli* (Ely and Pittard, 1979). Moreover, shikimate kinase is a target for the therapeutic inhibition of multi-resistant strains, including *K. pneumoniae* (Li et al., 2025). Overall, the functional pathways identified in this study highlight that *Enterobacteriaceae* and their metabolic functions contribute to the pathogenesis of NEC.

The limitations of this study include its single-center design and small sample size. However, to our knowledge, this is the first study in India to use a shotgun metagenomic approach to investigate the association of gut microbiota in fecal samples of infants with NEC.

## Conclusion

*Enterobacteriaceae* were more abundant in stool samples from infants with NEC than infants without NEC. Differential abundance analysis using Linear Discriminant Analysis Effect Size (LEfSe) identified *Klebsiella pneumoniae* and *Escherichia coli* as enriched in the gut microbiota of preterm infants with NEC. Functional analysis revealed increased expression of genes associated with the LPS O-antigen, the Type IV secretion system, the L-rhamnose pathway, quorum sensing, and iron transporters, including ABC transporters, in the NEC samples.

## Data Availability

The datasets presented in this study can be found in online repositories. The names of the repository/repositories and accession number(s) can be found below: NCBI–PRJNA1304237.

## References

[B1] Alsaied A. Islam N. Thalib L. (2020). Global incidence of Necrotizing Enterocolitis: a systematic review and Meta-analysis. BMC Pediatr. 20, 344. doi: 10.1186/s12887-020-02231-5, PMID: 32660457 PMC7359006

[B2] Brower-Sinning R. Zhong D. Good M. Firek B. Baker R. Sodhi C. P.. (2014). Mucosa-associated bacterial diversity in necrotizing enterocolitis. PloS One 9, e105046. doi: 10.1371/journal.pone.0105046, PMID: 25203729 PMC4159227

[B3] Cao Y. Dong Q. Wang D. Zhang P. Liu Y. Niu C. (2022). microbiomeMarker: an R/Bioconductor package for microbiome marker identification and visualization. Bioinformatics 38, 4027–4029. doi: 10.1093/bioinformatics/btac438, PMID: 35771644

[B4] Chen Y. Brook T. C. Soe C. Z. O’Neill I. Alcon-Giner C. Leelastwattanagul O.. (2020). Preterm infants harbour diverse Klebsiella populations, including atypical species that encode and produce an array of antimicrobial resistance- and virulence-associated factors. Microbial Genomics 6, e000377. doi: 10.1099/mgen.0.000377, PMID: 32436839 PMC7371107

[B5] Crellen T. Turner P. Pol S. Baker S. Nguyen T. N.T. Stoesser N.. (2019). “Transmission dynamics and control of multidrug-resistant Klebsiella pneumoniae in neonates in a developing country,” in eLife. (Cambridge CB2 1AW, UK: eLife Sciences Publications, Ltd). Available online at: https://elifesciences.org/articles/50468., PMID: 10.7554/eLife.50468 PMC6977969 31793878

[B6] Devarajalu P. Kumar J. Dutta S. Attri S. V. Kabeerdoss J. (2024). Gut microbiota of preterm infants in the neonatal intensive care unit: a study from a tertiary care center in northern India. Front. Microbiol. 15. doi: 10.3389/fmicb.2024.1329926, PMID: 38389529 PMC10881769

[B7] Devarajalu P. Kumar J. Dutta S. Attri S. V. Kabeerdoss J. (2025). Gut microbiota alteration in healthy preterm infants: an observational study from tertiary care center in India. Microorganisms 13, 577. doi: 10.3390/microorganisms13030577, PMID: 40142471 PMC11944540

[B8] Ely B. Pittard J. (1979). Aromatic amino acid biosynthesis: regulation of shikimate kinase in Escherichia coli K-12. J. Bacteriol 138, 933–943. doi: 10.1128/jb.138.3.933-943.1979, PMID: 222728 PMC218124

[B9] Huson D. H. Beier S. Flade I. Górska A. El-Hadidi M. Mitra S.. (2016). MEGAN community edition - interactive exploration and analysis of large-scale microbiome sequencing data. PloS Comput. Biol. 12, e1004957. doi: 10.1371/journal.pcbi.1004957, PMID: 27327495 PMC4915700

[B10] Li Y. Kumar S. Zhang L. (2025). Shikimate kinase 1 from klebsiella pneumoniae as a new drug target enzyme: insights from comparative modeling and molecular dynamics. Indian J. Pharm. Educ. Res. 58, 1110–1120. doi: 10.5530/ijper.58.4.122

[B11] Magnusson A. Jabbari Shiadeh S. M. Ardalan M. Swolin-Eide D. Elfvin A. (2024). Gut microbiota differences in five-year-old children that were born preterm with a history of necrotizing enterocolitis: A pilot trial. iScience 27, 110325. doi: 10.1016/j.isci.2024.110325, PMID: 39055941 PMC11269947

[B12] Mallick H. Rahnavard A. McIver L. J. Ma S. Zhang Y. Nguyen L. H.. (2021). Multivariable association discovery in population-scale meta-omics studies. PloS Comput. Biol. 17, e1009442. doi: 10.1371/journal.pcbi.1009442, PMID: 34784344 PMC8714082

[B13] Mara M. A. Good M. Weitkamp J.-H. (2018). Innate and adaptive immunity in necrotizing enterocolitis. Semin. Fetal Neonatal Med. 23, 394–399. doi: 10.1016/j.siny.2018.08.002, PMID: 30146477 PMC6269198

[B14] Marolda C. L. Valvano M. A. (1995). Genetic analysis of the dTDP-rhamnose biosynthesis region of the Escherichia coli VW187 (O7:K1) rfb gene cluster: identification of functional homologs of rfbB and rfbA in the rff cluster and correct location of the rffE gene. J. Bacteriol 177, 5539–5546. doi: 10.1128/jb.177.19.5539-5546.1995, PMID: 7559340 PMC177362

[B15] Mathers A. J. Crook D. Vaughan A. Barry K. E. Vegesana K. Stoesser N.. (2019). Klebsiella quasipneumoniae Provides a Window into Carbapenemase Gene Transfer, Plasmid Rearrangements, and Patient Interactions with the Hospital Environment. Antimicrobial Agents Chemotherapy 63, e02513-18. doi: 10.1128/aac.02513-18, PMID: 30910889 PMC6535554

[B16] McMurdie P. J. Holmes S. (2013). phyloseq: an R package for reproducible interactive analysis and graphics of microbiome census data. PloS One 8, e61217. doi: 10.1371/journal.pone.0061217, PMID: 23630581 PMC3632530

[B17] Millar M. R. MacKay P. Levene M. Langdale V. Martin C. (1992). Enterobacteriaceae and neonatal necrotising enterocolitis. Arch. Dis. Child 67, 53–56. doi: 10.1136/adc.67.1_spec_no.53, PMID: 1536588 PMC1590331

[B18] Morrow A. L. Lagomarcino A. J. Schibler K. R. Taft D. H. Yu Z. Wang B.. (2013). Early microbial and metabolomic signatures predict later onset of necrotizing enterocolitis in preterm infants. Microbiome 1, 13. doi: 10.1186/2049-2618-1-13, PMID: 24450576 PMC3971624

[B19] Oksanen J. Simpson G. L. Blanchet F. G. Kindt R. Legendre P. Minchin P. R.. (2001). vegan: Community Ecology Package. 2.6-8. doi: 10.32614/CRAN.package.vegan

[B20] Pammi M. Cope J. Tarr P. I. Warner B. B. Morrow A. L. Mai V.. (2017). Intestinal dysbiosis in preterm infants preceding necrotizing enterocolitis: a systematic review and meta-analysis. Microbiome 5, 31. doi: 10.1186/s40168-017-0248-8, PMID: 28274256 PMC5343300

[B21] Parks D. H. Tyson G. W. Hugenholtz P. Beiko R. G. (2014). STAMP: statistical analysis of taxonomic and functional profiles. Bioinformatics 30, 3123–3124. doi: 10.1093/bioinformatics/btu494, PMID: 25061070 PMC4609014

[B22] Paveglio S. Ledala N. Rezaul K. Lin Q. Zhou Y. Provatas A. A.. (2020). Cytotoxin-producing Klebsiella oxytoca in the preterm gut and its association with necrotizing enterocolitis. Emerg. Microbes Infect. 9, 1321–1329. doi: 10.1080/22221751.2020.1773743, PMID: 32525754 PMC7473113

[B23] Peschel S. Müller C. L. von Mutius E. Boulesteix A.-L. Depner M. Boulesteix A.-L. Depner M. (2021). NetCoMi: network construction and comparison for microbiome data in R. Briefings Bioinf. 22, bbaa290. doi: 10.1093/bib/bbaa290, PMID: 33264391 PMC8293835

[B24] Samara J. Moossavi S. Alshaikh B. Ortega V. A. Pettersen V. K. Ferdous T.. (2022). Supplementation with a probiotic mixture accelerates gut microbiome maturation and reduces intestinal inflammation in extremely preterm infants. Cell Host Microbe 30, 696–711.e5. doi: 10.1016/j.chom.2022.04.005, PMID: 35550672

[B25] Shaw A. G. Sim K. Rose G. Wooldridge D. J. Li M.-S. Misra R. V.. (2021). Premature neonatal gut microbial community patterns supporting an epithelial TLR-mediated pathway for necrotizing enterocolitis. BMC Microbiol. 21, 225. doi: 10.1186/s12866-021-02285-0, PMID: 34362295 PMC8343889

[B26] Tarracchini C. Milani C. Longhi G. Fontana F. Mancabelli L. Pintus R.. (2021). Unraveling the microbiome of necrotizing enterocolitis: insights in novel microbial and metabolomic biomarkers. Microbiol. Spectr. 9, e01176–e01121. doi: 10.1128/Spectrum.01176-21, PMID: 34704805 PMC8549755

[B27] Thänert R. Keen E. C. Dantas G. Warner B. B. Tarr P. I. (2020). Necrotizing enterocolitis and the microbiome: current status and future directions. J. Infect. Dis. 223, S257–S263. doi: 10.1093/infdis/jiaa604, PMID: 33330904 PMC8206796

[B28] Thänert R. Schwartz D. J. Keen E. C. Hall-Moore C. Wang B. Shaikh N.. (2024). Clinical sequelae of gut microbiome development and disruption in hospitalized preterm infants. Cell Host Microbe 32, 1822–1837.e5. doi: 10.1016/j.chom.2024.07.027, PMID: 39197454 PMC11466706

[B29] Wang Y. Jiang K. Xia Q. Kang X. Wang S. Yu J.-H.. (2024). Exploration of pathogenic microorganism within the small intestine of necrotizing enterocolitis. World J. Pediatr. 20, 165–172. doi: 10.1007/s12519-023-00756-0, PMID: 37676611

[B30] Ward D. V. Scholz M. Zolfo M. Taft D. H. Schibler K. R. Tett A.. (2016). Metagenomic sequencing with strain-level resolution implicates uropathogenic E. coli in necrotizing enterocolitis and mortality in preterm infants. Cell Rep. 14, 2912–2924. doi: 10.1016/j.celrep.2016.03.015, PMID: 26997279 PMC4819403

[B31] Zhou H. He K. Chen J. Zhang X. (2022). LinDA: linear models for differential abundance analysis of microbiome compositional data. Genome Biol. 23, 95. doi: 10.1186/s13059-022-02655-5, PMID: 35421994 PMC9012043

