# Correction: Characterization of gut microbiota signatures in Indian preterm infants with necrotizing enterocolitis: a shotgun metagenomic approach

**DOI:** 10.3389/fcimb.2025.1706582

**Published:** 2025-10-30

**Authors:** Prabavathi Devarajalu, Savita Verma Attri, Jogender Kumar, Sourabh Dutta, Jayakanthan Kabeerdoss

**Affiliations:** ^1^ Pediatric Biochemistry Unit, Department of Pediatrics, Post Graduate Institute of Medical Education & Research (PGIMER), Chandigarh, India; ^2^ Newborn Unit, Department of Pediatrics, Post Graduate Institute of Medical Education & Research (PGIMER), Chandigarh, India

**Keywords:** Necrotizing enterocolitis, gut microbiota, preterm infants, shotgun metagenomics, LPS O-antigen, TLR4, type IV secretion system (T4SS), India

There was a mistake in **Figure 4** as published. The incorrect **Figure 4**, ‘Heatmap showing three prominent genera of NEC and control groups’ was published. Figure 3b was mistakenly repeated as **Figure 4**. The corrected **Figure 4** appears below.

**Figure 4:**
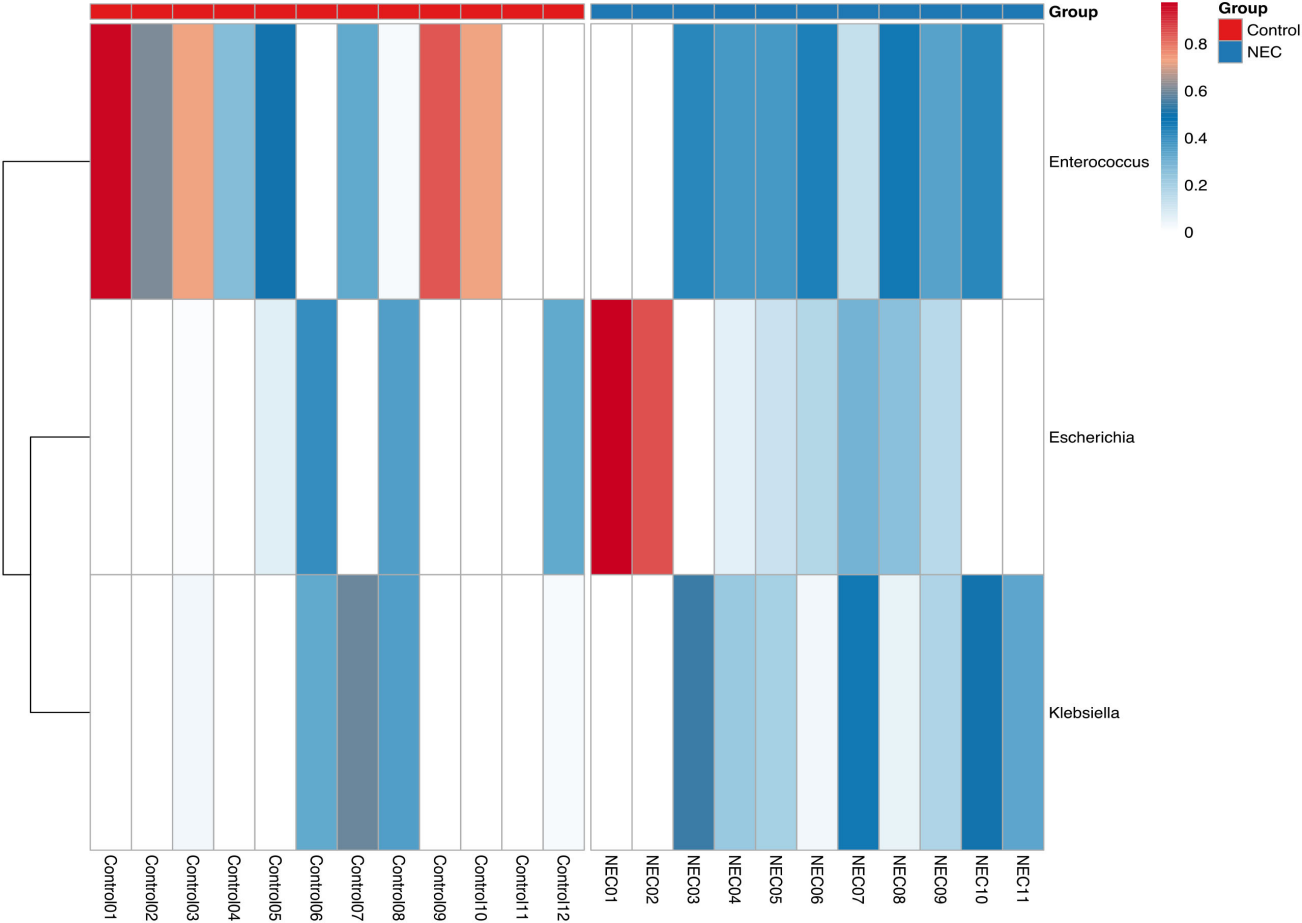
Heatmap showing three prominent genera of NEC and control groups.

The original version of this article has been updated.

