# Supplementary material for: Characterization of gut microbiota signatures in Indian preterm infants with necrotizing enterocolitis: a shotgun metagenomic approach
Source: Front Cell Infect Microbiol. 2025 Sep 11;15:1649384. doi: 10.3389/fcimb.2025.1649384 (PMC12460237; doi:10.3389/fcimb.2025.1649384)

**Table 1. Summary statistics of whole genome metagenome sequencing**

| **samples ID** | **Read orientation** | **Mean read quality (Phred score)** | **Number of reads** | **% Q > 30** | **Number of bases (MB)** | **Mean read length (bp)** |
| --- | --- | --- | --- | --- | --- | --- |
| Control01 | R1 | 39.15 | 25608087 | 95.89 | 3866.82 | 151 |
|  | R2 | 38.73 | 25608087 | 90.07 | 3866.82 | 151 |
| Control02 | R1 | 35.41 | 2317880 | 92.76 | 3500 | 151 |
|  | R2 | 35.73 | 23178808 | 94.14 | 3500 | 151 |
| Control03 | R1 | 38.9 | 26122716 | 94.86 | 3944.53 | 151 |
|  | R2 | 38.53 | 26122716 | 93.16 | 3944.53 | 151 |
| Control04 | R1 | 39.06 | 12940582 | 95.44 | 1954.03 | 151 |
|  | R2 | 38.47 | 12940582 | 92.91 | 1954.03 | 151 |
| Control05 | R1 | 39.02 | 26682439 | 95.35 | 4029.05 | 151 |
|  | R2 | 38.72 | 26682439 | 94.05 | 4029.05 | 151 |
| Control06 | R1 | 38.92 | 40118874 | 94.89 | 6057.95 | 151 |
|  | R2 | 38.23 | 40118874 | 91.89 | 6057.95 | 151 |
| Control07 | R1 | 35.81 | 16049887 | 93.19 | 2423.53 | 151 |
|  | R2 | 35.37 | 16049887 | 90.7 | 2423.53 | 151 |
| Control08 | R1 | 39.13 | 25830823 | 95.75 | 3900.45 | 151 |
|  | R2 | 38.69 | 25830823 | 93.78 | 3900.45 | 151 |
| Control09 | R1 | 35.22 | 11198143 | 90.45 | 1690.92 | 151 |
|  | R2 | 35.35 | 11198143 | 90.52 | 1690.92 | 151 |
| Control10 | R1 | 39.05 | 28291017 | 95.45 | 4271.94 | 151 |
|  | R2 | 38.71 | 28291017 | 93.94 | 93.94 | 151 |
| Control11 | R1 | 38.75 | 20828751 | 94.07 | 3145.14 | 151 |
|  | R2 | 37.72 | 20828751 | 89.55 | 3145.14 | 151 |
| Control12 | R1 | 38.97 | 25701711 | 95.14 | 3880.96 | 151 |
|  | R2 | 38.66 | 25701711 | 93.73 | 3880.96 | 151 |
| NEC01 | R1 | 38.89 | 26907246 | 94.81 | 4062.99 | 151 |
|  | R2 | 38.47 | 26907246 | 92.94 | 4062.99 | 151 |
| NEC02 | R1 | 38.8 | 28763256 | 94.43 | 4343.25 | 151 |
|  | R2 | 38.45 | 28763256 | 92.83 | 4343.25 | 151 |
| NEC03 | R1 | 34.42 | 23178808 | 88.07 | 3500 | 151 |
|  | R2 | 35.46 | 23178808 | 92.91 | 3500 | 151 |
| NEC04 | R1 | 38.43 | 23187060 | 92.65 | 3501.25 | 151 |
|  | R2 | 37.96 | 23187060 | 90.52 | 3501.25 | 151 |
| NEC05 | R1 | 38.76 | 28451325 | 94.24 | 4296.15 | 151 |
|  | R2 | 38.39 | 28451325 | 92.59 | 4296.15 | 151 |
| NEC06 | R1 | 38.88 | 13620540 | 94.75 | 2056.7 | 151 |
|  | R2 | 38.18 | 13620540 | 91.7 | 2056.7 | 151 |
| NEC07 | R1 | 38.9 | 30165498 | 94.8 | 4554.99 | 151 |
|  | R2 | 38.45 | 30165498 | 92.86 | 4554.99 | 151 |
| NEC08 | R1 | 35.76 | 17142097 | 92.99 | 2588.46 | 151 |
|  | R2 | 35.44 | 17142097 | 91.04 | 2588.46 | 151 |
| NEC09 | R1 | 35.79 | 22084318 | 93.07 | 3334.73 | 151 |
|  | R2 | 35.37 | 22084318 | 90.68 | 3334.73 | 151 |
| NEC10 | R1 | 38.6 | 18104100 | 93.4 | 2733.72 | 151 |
|  | R2 | 38.01 | 18104100 | 90.8 | 2733.72 | 151 |
| NEC11 | R1 | 35.74 | 21926438 | 92.85 | 3310.89 | 151 |
|  | R2 | 35.87 | 21926438 | 93.36 | 3310.89 | 151 |

**Supplementary figure 1. Correlation and network analyses were performed using the Network Construction and Comparison for Microbiome Data (NetCoMi) package**

1a. Correlation analysis


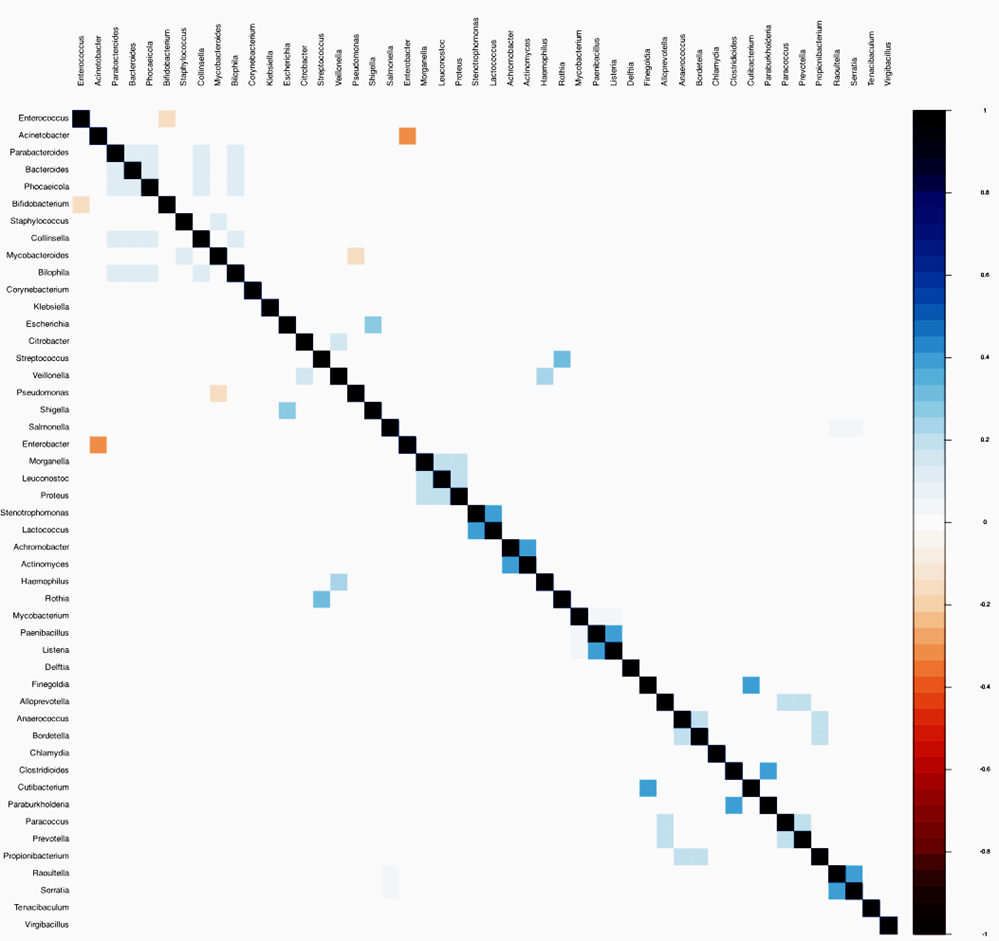


1b. Network analysis


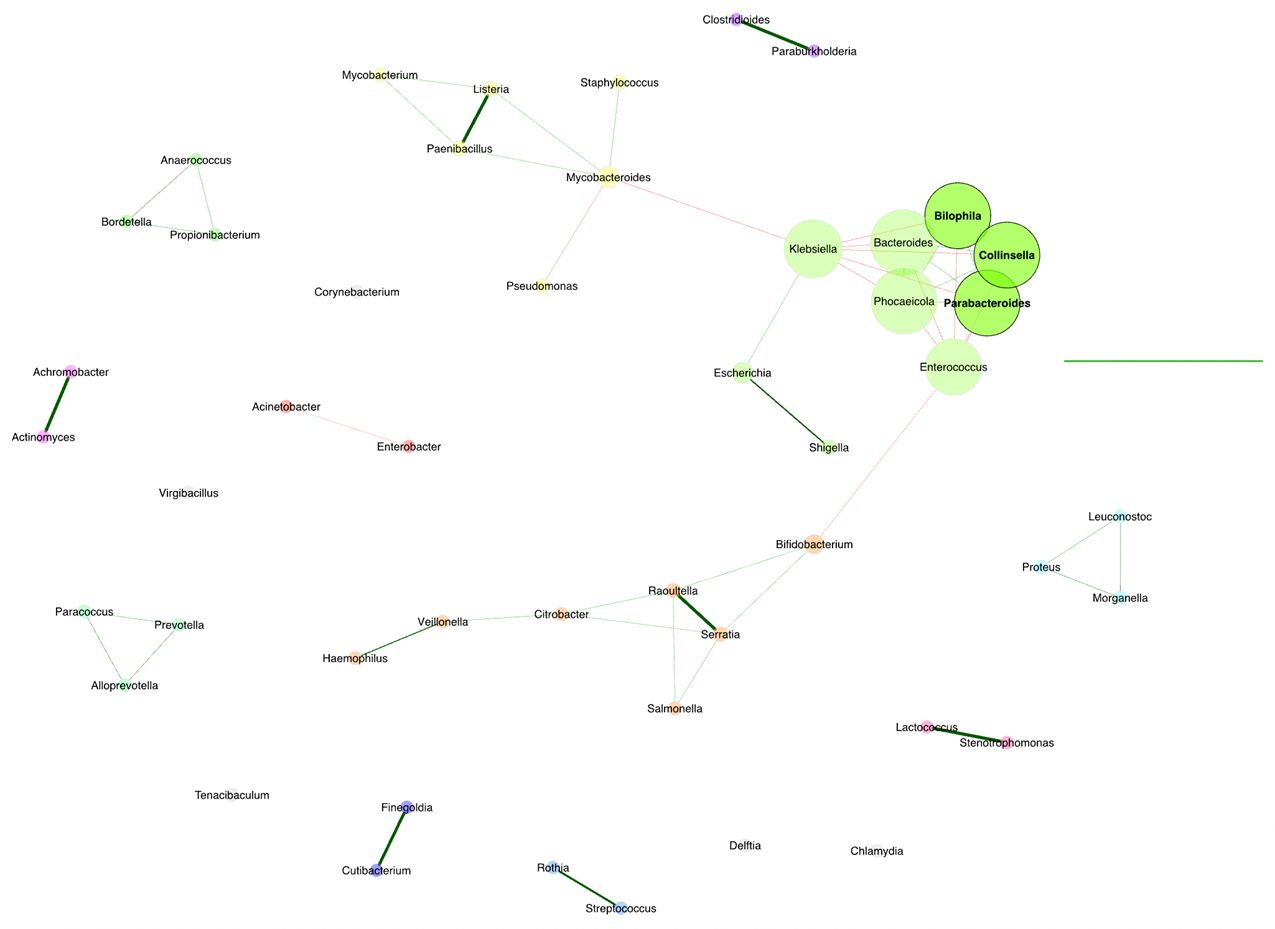


**Supplementary figure 2. Differential functional analysis performed between NEC and control using Welch’s test in STAMP tool.**


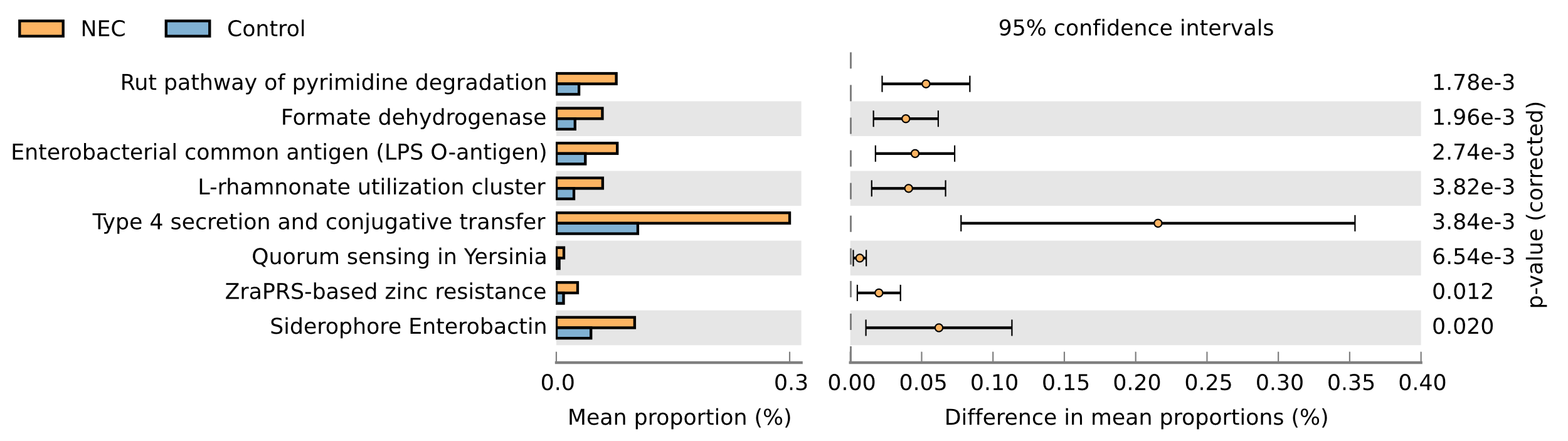

Supplement: Supplementary file 1 [file Table1.docx]
